# ZD6474 – a novel inhibitor of VEGFR and EGFR tyrosine kinase activity

**DOI:** 10.1038/sj.bjc.6602603

**Published:** 2005-05-31

**Authors:** A J Ryan, S R Wedge

**Affiliations:** 1 Department of Cancer and Infection Research, AstraZeneca, Macclesfield, Cheshire SK10 4TG, UK

**Keywords:** ZD6474, VEGFR inhibitor, EGFR inhibitor, antitumour, angiogenesis

## Abstract

Angiogenesis is crucial for maintaining the supply of oxygen and nutrients required to support solid tumour growth. Inhibitors of tumour blood vessel formation are therefore being sought, in particular, inhibitors of vascular endothelial growth factor-A (VEGF)-signalling, which has a pivotal role in stimulating neovascular growth and survival. ZD6474 is an orally bioavailable inhibitor of VEGF receptor-2 tyrosine kinase activity that in preclinical studies has been shown to inhibit both VEGF-induced signalling in endothelial cells and tumour-induced angiogenesis. Consistent with inhibition of angiogenesis, once-daily oral dosing of ZD6474 produced significant broad-spectrum antitumour activity in a panel of histologically diverse human tumour xenografts. In addition to its antiangiogenic properties, ZD6474 also has activity against the epidermal growth factor receptor (EGFR) tyrosine kinase, which could impart a direct inhibitory effect on tumour cell growth and survival. This may be particularly relevant in tumours with a dependency upon EGFR signalling, for example in certain tumours harbouring activating mutations in EGFR. RET kinase has also been identified as a third target for ZD6474. This review summarises preclinical studies with this unique agent and considers its future direction in cancer treatment.

In order to grow beyond a few millimetres in size, tumours must develop a functioning vascular network to provide essential nutrients and oxygen. Early in tumorigenesis, an ‘angiogenic switch’ occurs within the tumour microenviro nment, which is associated with the altered expression of a range of proangiogenic growth factors, such as vascular endothelial growth factor-A (VEGF) and basic fibroblast growth factor (bFGF). The resulting imbalance of proangiogenic factors stimulates the sprouting of new blood vessels from pre-existing vessels within the surrounding normal tissue. These new vessels grow and infiltrate into the tumour, providing nutrients and oxygen to sustain tumour expansion, and a route for cancer cell dissemination. The carefully controlled balance of pro- and antiangiogenic growth factors during physiological angiogenesis (wound healing, menstrual cycle and pregnancy) produces well-ordered vascular structures with mature, functional blood vessels. In contrast, pathological angiogenesis associated with tumour growth is characterised by disorganised vasculature that lacks clear hierarchical organisation, with associated abnormalities in blood flow, vascular permeability and pericyte–endothelial cell interactions, and with a high fraction of immature, proliferating endothelial cells.

While many factors have been implicated in promoting angiogenesis, VEGF appears to play a pivotal rate-limiting role, which has been attributed to its ability to regulate key steps in the angiogenesis cascade, including inducing endothelial cell proliferation, migration, survival and capillary tube formation (Benjamin *et al*, 1999; Yancopoulos *et al*, 2000; Jain, 2003; Ferrara, 2004). Vascular endothelial growth factor also induces increased vascular permeability, which is a characteristic of tumour neovasculature (Senger *et al*, 1983; Dvorak *et al*, 1995). Vascular endothelial growth factor mRNA is upregulated in human cancers, as a consequence of oncogene activation, loss of tumour suppressor function or hypoxia resulting from suboptimal perfusion (Shweiki *et al*, 1995; Rak *et al*, 2000). High levels of VEGF receptor (VEGFR) mRNA are also detectable in tumour-associated endothelial cells (Brown *et al*, 1993a, 1993b; Plate *et al*, 1994), and high circulating plasma levels of VEGF have been shown to correlate with poor prognosis in patients with a variety of solid malignancies (Poon *et al*, 2001; Jubb *et al*, 2004).

Vascular endothelial growth factor binds to the receptors VEGFR-1 (Flt-1) and VEGFR-2 (kinase insert domain-containing receptor; KDR) on vascular endothelial cells. Significantly, activation of VEGFR-2 alone is necessary and sufficient to effect VEGF-induced mitogenesis, angiogenesis and vascular permeability (Ferrara *et al*, 2003). Vascular endothelial growth factor receptors are single-pass transmembrane receptors that possess intrinsic receptor tyrosine kinase activity within the cytoplasmic domain of the receptor. Ligand (VEGF) binding results in VEGFR dimerisation, which in turn triggers kinase activation and autophosphorylation of specific intracellular VEGFR tyrosine residues. Autophosphorylation at these tyrosine residues further increases the catalytic activity of the tyrosine kinase, and provides potential docking sites for cytoplasmic signal transduction molecules such as PLC-γ (Matsumoto and Claesson-Welsh, 2001). These protein interactions are essential for mediating the intracellular signalling that is required to induce cellular responses to VEGF (for example, endothelial cell proliferation, survival and migration).

Recognition of the key role of VEGF-mediated VEGFR-2 signalling in pathological angiogenesis has led to the development of various targeted strategies designed to inhibit VEGFR-2 activation, which include ligand sequestration, receptor antagonist approaches or receptor tyrosine kinase inhibition (Madhusudan and Harris, 2002; Manley *et al*, 2002; Ferrara *et al*, 2003). Of these, the latter has been approached by the development of small molecule ATP-competitive inhibitors that are compatible with long-term oral administration. Although ATP binding is a common feature of the ∼518 protein kinases encoded by the human genome, a small molecule inhibitor may interact with nonconserved structural features within, or directly adjacent to, the ATP binding site to attain kinase selectivity. By obstructing ATP binding within a given kinase domain, the inhibitor prevents the subsequent autophosphorylation that is mandatory for initiating intracellular signal transduction (Noble *et al*, 2004).

ZD6474 is a novel heteroaromatic-substituted anilinoquinazoline (Figure 1) that acts as a potent and reversible inhibitor of ATP binding to VEGFR-2 tyrosine kinase. It was selected from a subseries of compounds for further development after demonstrating nanomolar inhibition of VEGFR-2 tyrosine kinase activity *in vitro*, combined with good oral bioavailability and sustained plasma drug levels supporting once-daily oral therapy *in vivo* (Hennequin *et al*, 2002).

In addition, ZD6474 also inhibits epidermal growth factor receptor (EGFR) tyrosine kinase activity. The EGFR autocrine signalling pathway is central to cancer progression, and overexpression of EGFR and/or its ligands, transforming growth factor (TGF)-α and EGF has been reported in many human tumours (Ciardiello and Tortora, 2001). The consequences of aberrant EGFR tyrosine kinase activity include increased tumour cell proliferation, survival and invasiveness (Salomon *et al*, 1995; Perrotte *et al*, 1999), as well as the overexpression of proangiogenic factors such as VEGF (Goldman *et al*, 1993; Gille *et al*, 1997). In addition, inhibition of EGFR signalling has been shown to induce selective apoptosis in tumour endothelial cells in an orthotopic model of human renal cell carcinoma metastasis in bone (Weber *et al*, 2003). It was postulated that some tumours may produce high levels of EGFR ligands (TGF-*α*, EGF), thus stimulating EGFR expression in tumour-associated endothelial cells and setting up a paracrine endothelial cell survival signalling pathway that is sensitive to inhibitors of EGFR signalling (Weber *et al*, 2003).

Consequently, ZD6474 has the potential to inhibit two key pathways in tumour growth by (i) targeting tumour growth indirectly, via inhibition of VEGF-dependent tumour angiogenesis and VEGF-dependent endothelial cell survival, and (ii) targeting tumour growth directly, via inhibition of EGFR-dependent tumour cell proliferation and survival (Figure 1).

## *IN VITRO* PRECLINICAL EVALUATION

### Selective targeting of VEGFR and EGFR tyrosine kinase activity

The ability of ZD6474 to inhibit tyrosine kinase activity was determined using recombinant enzyme assays (Table 1; Wedge *et al*, 2002). These studies identified ZD6474 as a potent inhibitor of VEGFR-2 kinase activity (IC_50_=40 nM). Compared with the IC_50_ for VEGFR-2 kinase, ZD6474 was only 2.7-fold less active *vs* VEGFR-3 (Flt-4) kinase, but demonstrated 40-fold selectivity *vs* the kinase associated with VEGFR-1. Vascular endothelial growth factor receptor-3 and its ligands (VEGF-C and VEGF-D) are known to play key roles in the regulation of lymphangiogenesis (Jussila and Alitalo, 2002). Although thought to be largely restricted to lymphatic endothelium, VEGFR-3 expression has recently been detected on the vascular endothelium of human tumours, most notably in renal cell carcinoma, although the functional significance of this remains to be determined (Bando *et al*, 2004). In addition, there is emerging evidence from animal models that VEGF-C overexpression may promote metastatic spread to lymph nodes (Schietroma *et al*, 2003) and clinically, high expression of VEGF-C and/or -D has been found to predict for nodal metastasis and poor prognosis in a number of different tumour types (Kurahara *et al*, 2004; Zeng *et al*, 2004; Suzuki *et al*, 2005). Determining the relevance of ZD6474 inhibition of VEGFR-3 kinase activity in recombinant enzyme assays requires further evaluation of receptor signalling in cellular assays.

ZD6474 was also shown to inhibit EGFR tyrosine kinase activity in recombinant enzyme assays (IC_50_=500 nM), but demonstrated excellent selectivity for VEGFR-2 kinase *vs* structurally related receptor tyrosine kinases, such as c-kit, and kinases from other families (Table 1).

Consistent with its activity *vs* isolated VEGFR-2, ZD6474 is a potent inhibitor of VEGF-stimulated human umbilical vein endothelial cell (HUVEC) proliferation *in vitro* (IC_50_=60 nM) (Wedge *et al*, 2002). In assays measuring inhibition of EGF-stimulated HUVEC proliferation, ZD6474 was approximately three-fold less potent (IC_50_=170 nM). In contrast, much higher doses of ZD6474 were required to inhibit bFGF-stimulated HUVEC proliferation (IC_50_=800 nM) or basal (serum-stimulated) HUVEC proliferation (IC_50_>3000 nM). These data demonstrate that ZD6474 is a selective inhibitor of VEGF- and EGF-stimulated cell proliferation.

### Direct inhibition of tumour cell growth – influence of EGFR mutation

The primary antitumour effect of ZD6474 is considered to be via inhibition of VEGFR tyrosine kinase activity in tumour endothelial cells. However, *in vitro* evidence shows that ZD6474 can also elicit direct inhibition of tumour cell growth (Arao *et al*, 2004). ZD6474 inhibited the growth of cell lines derived from various human cancers, including lung, breast, ovarian and colon cancer. This effect appeared to be via inhibition of EGFR tyrosine kinase activity, since the relative IC_50_ values of ZD6474 matched those of gefitinib, a specific inhibitor of EGFR tyrosine kinase. Among the cell lines examined, one derived from a Japanese female patient with adenocarcinoma of the lung (PC-9) was shown to be hypersensitive to ZD6474 (IC_50_=90 nM for serum-stimulated tumour cell growth). PC-9 has a 15 bp deletion mutation in the gene encoding the EGFR. The EGFR tyrosine kinase of this mutated gene is active even in the absence of exogenously added ligand, and is more sensitive to the inhibitory effects of ZD6474 than wild-type EGFR tyrosine kinase (Arao *et al*, 2004; Taguchi *et al*, 2004). Overall, these results suggest that ZD6474 can inhibit EGFR-dependent tumour growth, and also that the magnitude of inhibition may depend upon the expression of mutant EGFR.

### Additional activity *vs* RET kinase

ZD6474 has also demonstrated potent inhibition of ligand-dependent RET receptor tyrosine kinase activity (IC_50_=100 nM) and selective inhibition of RET-dependent thyroid tumour cell growth *in vitro* (Carlomagno *et al*, 2002; Carlomagno *et al*, 2004). ZD6474 inhibited the majority of mutated, activated forms of RET receptor tyrosine kinase and also inhibited the wild-type enzyme. Therefore, in addition to inhibition of VEGFR-2 tyrosine kinase and EGFR tyrosine kinase, inhibition of RET tyrosine kinase by ZD6474 may provide particular additional antitumour effects in the treatment of tumours with genetic changes in the *RET* gene (mutation or translocation) that lead to RET receptor signalling-dependent tumour cell growth (Santoro *et al*, 2002).

## *IN VIVO* PRECLINICAL EVALUATION

### ZD6474 inhibits VEGF signalling, angiogenesis and vascular permeability *in vivo*

The ability of ZD6474 to inhibit VEGF signalling selectively *in vivo* was demonstrated in a hypotension assay in anaesthetised rat, where a number of growth factors are known to induce acute hypotensive changes by signalling through their cognate receptor. In this model, ZD6474 showed reversal of hypotension induced by VEGF, but did not significantly reverse hypotension induced by bFGF (Wedge *et al*, 2002).

The effects of ZD6474 on epiphyseal growth-plate morphology and ossification were also examined, since this is a physiological process critically dependent upon angiogenic invasion into the growth plate and VEGF-signalling within cellular subtypes to regulate bone morphogenesis. Consistent with inhibition of VEGF signalling, ZD6474 treatment inhibited ossification and produced a dose-dependent increase in epiphyseal growth-plate hypertrophy (Wedge *et al*, 2002).

**Figure 2:**
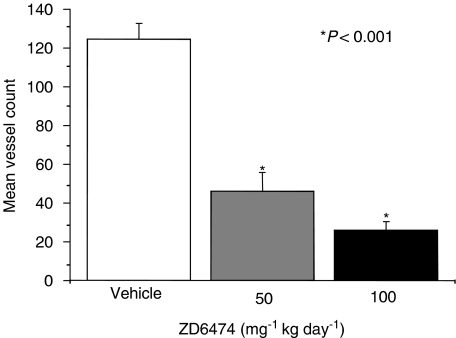
ZD6474 inhibits tumour-induced new blood vessel development. A549 tumour cells were implanted intradermally and new blood vessel formation was assessed after 5 days' administration of ZD6474 (50 or 100 mg kg^−1^ day^−1^) or vehicle (Wedge *et al*, 2002).

**Figure 3:**
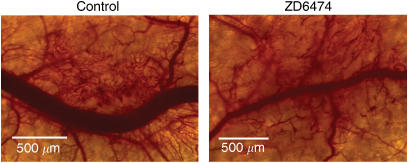
ZD6474 reduces tumour microvessel density. Intravital microscopy image taken in mice with L3.6pl pancreatic tumours growing in dorsal skin-fold chambers after 6 days' treatment with ZD6474 (50 mg kg^−1^ day^−1^) (Figure provided by CJ Bruns).

Several preclinical studies have shown that ZD6474 inhibits tumour angiogenesis *in vivo.* ZD6474 significantly inhibited new blood vessel formation following intradermal transplantation of human non-small-cell lung cancer (NSCLC) cells (Figure 2; Wedge *et al*, 2002), and tumour microvessel density was reduced by ZD6474 administration in orthotopic models of human gastric (McCarty *et al*, 2004) and pancreatic cancer (Figure 3; Bruns *et al*, 2003). A striking observation in the gastric cancer model was the increased proportion of pericyte-associated endothelial cells post-ZD6474 treatment, despite a marked reduction in the overall number of tumour endothelial cells. This finding is likely to be due to an effect of ZD6474 on the survival of tumour blood vessels that are not stabilised by perivascular support. A similar finding was reported in human prostate cancer, with selective apoptosis of perictye-free endothelial cells occurring after hormone-therapy-induced loss of VEGF activity (Benjamin *et al*, 1999). Immature blood vessels are known to be reliant on VEGF-induced survival signalling, probably mediated by the PI3K/AKT pathway (Gerber *et al*, 1998).

Dynamic contrast-enhanced magnetic resonance imaging (DCE-MRI) is a technique that can be utilised to measure tumour haemodynamic parameters *in vivo*. DCE-MRI may therefore be used to detect alterations in tumour vascular permeability and/or perfusion in response to drug treatment. This technology is currently being evaluated in clinical trials with VEGF signalling inhibitors, since VEGF is known to enhance vascular permeability significantly (Morgan *et al*, 2003; Willett *et al*, 2004). Encouraging preclinical results have been obtained with ZD6474 in mouse models of human prostate (Checkley *et al*, 2003) and colon (Bradley *et al*, 2004) cancer. In both studies, DCE-MRI analysis showed a dose-dependent reduction in contrast agent uptake by tumours, 24 h after initiating therapy with ZD6474. These results are consistent with ZD6474 inhibition of VEGF-induced permeability in tumour vasculature (Dvorak *et al*, 1995).

### ZD6474 inhibits tumour growth in a wide range of models

**Figure 4:**
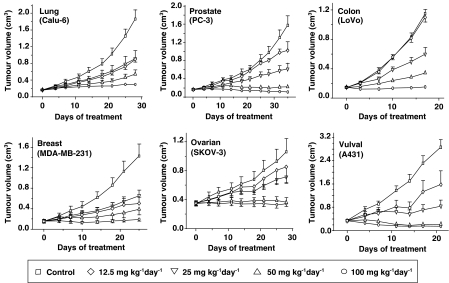
Antitumour efficacy of ZD6474 in a range of tumour types (adapted from Wedge *et al*, 2002).

ZD6474 has demonstrated broad-spectrum antitumour activity in preclinical models. In a histologically diverse range of tumour xenograft models (lung, prostate, breast, ovarian, colon or vulval), chronic once-daily oral administration of ZD6474 at doses of 25−100 mg kg^−1^ produced significant, dose-dependent inhibition of tumour growth (Figure 4; Wedge *et al*, 2002). Drug treatment was well tolerated at doses of 100 mg kg^−1^ day^−1^ for up to 5 weeks. The broad-spectrum activity observed with ZD6474 in tumour models is consistent with inhibition of VEGF signalling and an indirect (that is, antiangiogenic) antitumour effect rather than direct antiproliferative effects on the tumour cell. To support this, we have shown that tumour models that are known to be either sensitive (for example, A431 and LoVo) or insensitive (for example, PC-3 and Calu-6) to the selective EGFR tyrosine kinase inhibitor gefitinib, are all sensitive to ZD6474 (Figure 4).

ZD6474 also displayed antitumour activity in a xenograft model with acquired resistance to EGFR signalling inhibitors (Ciardiello *et al*, 2004). The authors attributed this effect of ZD6474 to inhibition of VEGF signalling, since *in vitro* data indicated that VEGF expression was upregulated in EGFR inhibitor-resistant cells. Gefitinib-resistant tumour cells were crossresistant to ZD6474 *in vitro*, but not *in vivo*. This suggests that in these tumour cells the *in vitro* antitumour activity of ZD6474 is dependent on EGFR tyrosine kinase inhibition, whereas the *in vivo* antitumour activity of ZD6474 is not. Collectively, these data suggest that ZD6474 may be an effective treatment against tumours with acquired or intrinsic EGFR resistance, because of its ability to inhibit VEGF signalling. Nonetheless, additional inhibition of EGFR tyrosine kinase may afford further therapeutic benefits, depending upon the tumour type. To date, this has been most profoundly shown in a preclinical *in vivo* study examining established PC-9 human lung cancer xenografts. ZD6474 (12.5–50 mg kg^−1^ day^−1^) caused robust *regression* of PC-9 tumours at all doses (Taguchi *et al*, 2004). As described previously, PC-9 tumour cells harbour an activating mutation of the EGFR gene and are highly sensitive to ZD6474 treatment *in vitro*. Therefore, in tumour cells that are highly dependent on EGFR signalling for continued proliferation and survival, direct tumour cell effects may contribute significantly to the *in vivo* antitumour activity of ZD6474.

ZD6474 has also been shown to inhibit growth of tumours implanted orthotopically (that is, implantation at the tissue/organ site from which the tumour originated). Organ–tumour interactions at the site of the primary tumour and at the site(s) of metastasis are considered important determinants of tumour growth and development in man. Therefore, it has been suggested that orthotopically implanted tumours may recapitulate the natural tumour setting more accurately than subcutaneous models (Taghian and Suit, 1999). In orthotopic models of gastric and pancreatic cancer, ZD6474 administration significantly inhibited tumour growth and increased tumour cell apoptosis (Bruns *et al*, 2003; McCarty *et al*, 2004). Almost complete suppression of tumour growth and angiogenesis was also observed with ZD6474 treatment in orthotopic models of lung cancer (Wu *et al*, 2003, 2004).

The effect of ZD6474 in early tumorigenesis has been investigated further in multiple intestinal neoplasia (*Min*) mice, which develop spontaneous early intestinal adenomas (Wilkinson *et al*, 2004). ZD6474 treatment (50 mg kg^−1^ day^−1^ for 4 weeks) produced a 75% reduction in overall tumour burden compared with vehicle treated controls (18.3±5.6 *vs* 76.5±11.7 mm^3^, *P*=0.005), suggesting that ZD6474 could be an effective treatment for early-stage disease. Indeed, early administration of ZD6474 has also been shown to significantly inhibit mammary tumour formation in a 7,12-dimethylbenz[a]anthracene model of breast cancer in Sprague–Dawley rats (Heffelfinger *et al*, 2004).

### ZD6474 inhibits tumour metastasis

The growth of both primary tumours and metastases are dependent on the development of new blood vessels (Folkman, 2002). In addition, the aberrant, disorganised tumour vasculature provides a route whereby tumour cells can escape the primary tumour mass and disseminate to distant metastatic sites. In a model of liver metastasis, ZD6474 treatment resulted in a significant reduction in metastatic blood flow and a diminished metastatic burden (Varghese *et al*, 2003). ZD6474 (50 mg kg^−1^ day^−1^) has also been shown to inhibit the production of lung metastases in an orthotopic model of renal cell carcinoma (Drevs *et al*, 2005). Similarly, lung and lymph node metastases were seen to be reduced following ZD6474 treatment in a metastatic human pancreatic tumour model (Bruns *et al*, 2003). While speculative, it is not inconceivable that ZD6474 activity *vs* VEGFR-3 tyrosine kinase could have contributed to the observed reduction in lymphatic metastasis, particularly since approaches that selectively inhibit activation of either VEGFR-2 or EGFR did not inhibit lymph node metastases significantly in this model (Bruns *et al*, 2000, 2002). Daily oral dosing with ZD6474 has also been found to inhibit metastasis from established lung tumours (Wu *et al*, 2004) and to limit the growth of NSCLC metastases (Matsumori *et al*, 2003). Overall, these data demonstrate that ZD6474 has the potential to inhibit metastasis by prevention of primary tumour dissemination, as well as by inhibiting the growth of any secondary tumours that become established.

### ZD6474 in combination with other anticancer therapies

**Figure 5:**
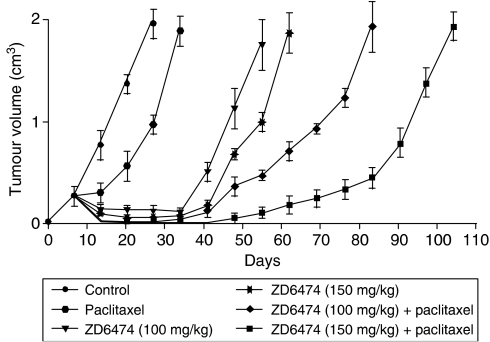
Antitumour activity of ZD6474 in combination with paclitaxel on established GEO xenografts. After inoculation with GEO cells, mice were treated on days 1–5 of each week for 4 weeks with ZD6474 (100 or 150 mg kg^−1^ day^−1^), alone or in combination with paclitaxel (20 mg kg^−1^ dose^−1^) on day 1 of each week for 4 weeks (Ciardiello *et al*, 2003).

The results of a number of preclinical studies suggest that combining ZD6474 with certain anticancer strategies may yield additional therapeutic benefits. In a xenograft model of colon cancer, ZD6474 (100 or 150 mg kg^−1^ dose^−1^) combined with the conventional cytotoxic agent paclitaxel (20 mg kg^−1^ dose^−1^) produced a greater inhibition of tumour growth than that seen with either agent alone (Figure 5; Ciardiello *et al*, 2003).

Studies have also investigated the efficacy of combining ZD6474 with radiotherapy *in vivo*. Vascular endothelial growth factor is an important survival factor for endothelial cells, and inhibition of VEGF signalling may augment the effects of ionizing radiation by preventing VEGF-mediated recovery of damaged endothelial cells (Gorski *et al*, 1999). Concurrent administration of ZD6474 and radiation therapy in two xenograft models of lung cancer showed greater antitumour effects compared with radiation therapy alone (Hoang *et al*, 2004). The impact of scheduling ZD6474 and radiation therapy has been investigated in Calu-6 xenografts (Williams *et al*, 2004). Any inhibitory effect of ZD6474 in this tumour model can be considered to be a function of its anti-VEGFR activity, since tumour growth was unresponsive to a specific inhibitor of EGFR tyrosine kinase. The study compared the effects of radiotherapy in combination with concurrent or sequential ZD6474 administration. Both combination schedules significantly enhanced treatment efficacy compared with radiotherapy or ZD6474 alone, but administration of ZD6474 after radiotherapy was more effective than concurrent treatment. Irradiation is known to cause transient tumour hypoxia, which in turn induces VEGF expression (Gorski *et al*, 1999). The greater efficacy observed in combination may possibly be attributed to ZD6474 inhibiting the VEGF-dependent survival of endothelial cells when administered after radiotherapy.

ZD6474 has also been examined in combination with SC-236, a selective cyclooxygenase-2 (COX-2) inhibitor, in human xenograft models of lung and colon cancer (Tuccillo *et al*, 2005). Overexpression of COX-2 has been linked with promotion of tumour cell growth and angiogenesis in human cancer (Masferrer *et al*, 2000; Masunaga *et al*, 2000). Combined ZD6474 and SC-236 treatment caused a supra-additive inhibition of established tumour growth in both models, further demonstrating the potential for ZD6474 to act in concert with other cancer treatments.

## CONCLUSIONS AND FUTURE PERSPECTIVES

ZD6474 is a potent inhibitor of VEGFR-2 tyrosine kinase activity and has demonstrated antitumour effects in a range of subcutaneous xenograft, orthotopic and metastatic preclinical tumour models, including those with intrinsic or acquired EGFR resistance. This spectrum of activity is consistent with an antiangiogenic mode of action, mediated through inhibition of VEGFR tyrosine kinase activity in tumour-associated endothelial cells. The clinical value of VEGF inhibition in the treatment of cancer has already been demonstrated unequivocally with bevacizumab, an anti-VEGF monoclonal antibody, when used in combination with conventional chemotherapy in patients with colorectal cancer (Hurwitz *et al*, 2004). ZD6474 may also benefit from inhibiting EGFR tyrosine kinase in tumour cells with a dependency upon EGFR signalling for survival or proliferation. That inhibition of both VEGFR and EGFR signalling may contribute to the net antitumour activity of ZD6474 represents an interesting and unique profile amongst molecular targeted therapies.

Achieving an optimal clinical response to ZD6474 may depend on several factors. These include its use in combination with other anticancer strategies and the stage of tumour development when treatment is initiated. *In vivo* preclinical studies indicate that the antitumour effects of ZD6474 are enhanced when it is used in combination with certain chemotherapies. This is being investigated further in ongoing phase II clinical trials of ZD6474, in which patients with a range of tumour types will receive ZD6474 alone or in combination with certain conventional cytotoxic agents. Promising preclinical results have been obtained using ZD6474 in combination with radiotherapy, with the important caveat that optimal efficacy may be dependent upon the schedule of administration. Preliminary evidence is also emerging that suggests tumour susceptibility to ZD6474 may be influenced by mutations in the biological targets within the tumour (EGFR, RET). Further mutational analysis of both EGFR and RET may be necessary to better understand the clinical significance of this phenomenon.

The activity of ZD6474 against RET kinase provides an opportunity to target specific patient subsets who may obtain particular benefit from this pharmacological activity. The *RET* gene can be activated by somatic rearrangements in papillary thyroid carcinoma or by point mutations in sporadic and familial (MEN2) medullary thyroid carcinomas. Therefore, ZD6474 might be especially beneficial in the treatment of thyroid tumours with known mutations in the *RET* gene (Santoro *et al*, 2002). In these cases, the combined effect of targeting RET and EGFR in the thyroid cancer cells, and targeting VEGFR-2 in the tumour vasculature may provide distinct advantages, and offer a focused route for translating the unique profile of this agent into a meaningful treatment for patients.

In conclusion, ZD6474 is a potent, orally bioavailable inhibitor of VEGFR and EGFR tyrosine kinase activity. It is a novel molecular-targeted agent that has shown promising antitumour activity in preclinical models. Given early reports of clinical activity with this agent in NSCLC (Minami *et al*, 2003), results from additional clinical studies that will evaluate ZD6474 either as monotherapy or in combination with established therapeutic modalities, are eagerly awaited.

## Figures and Tables

**Figure 1:**
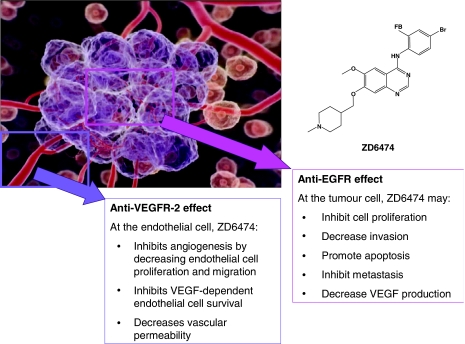
ZD6474 chemical structure and mechanism of action.

**Table 1:** ZD6474 kinase selectivity (adapted from Wedge *et al*, 2002)

**Kinase**	**IC_50_ (*μ*M)**
VEGFR-2 (KDR)	0.04
VEGFR-3 (Flt-4)	0.11
RET	0.13
EGFR	0.5
	
VEGFR-1 (Flt-1)	>1
PDGFR-*β*	>1
Tie-2	>1
FGFR1	>1
MEK	>10
CDK2	>10
c-kit	>20
erbB2	>20
FAK	>20
PDK1	>20
AKT	>100
IGF-1R	>200
